# Critical appraisal beyond clinical guidelines for intraabdominal candidiasis

**DOI:** 10.1186/s13054-023-04673-6

**Published:** 2023-10-03

**Authors:** Emilio Maseda, Ignacio Martín-Loeches, Rafael Zaragoza, Javier Pemán, Jesús Fortún, Santiago Grau, Gerardo Aguilar, Marina Varela, Marcio Borges, María-José Giménez, Alejandro Rodríguez

**Affiliations:** 1 Service of Anesthesia, Hospital Quirónsalud Valle del Henares, Av. de La Constitución, 249, 28850 Torrejón de Ardoz, Madrid, Spain; 2 Department of Intensive Care Medicine, Multidisciplinary Intensive Care Research Organization (MICRO), St James’s Hospital, James Street, Leinster, Dublin 8, D08 NHY1 Ireland; 3 Pulmonary Intensive Care Unit, Respiratory Institute, Hospital Clinic of Barcelona, IDIBAPS (Institut d’Investigacions Biomèdiques August Pi I Sunyer), University of Barcelona, CIBERes, Barcelona, Spain; 4 ICU, Hospital Universitario Dr. Peset, Valencia, Spain; 5 Microbiology Department, Hospital Universitari I Politecnic La Fe, Valencia, Spain; 6 Infectious Diseases Service, Hospital Universitario Ramón y Cajal, Madrid, Spain; 7 Service of Pharmacy, Hospital del Mar, Barcelona, Spain; 8 Service of Anesthesia, Hospital Clínico Universitario de Valencia, Valencia, Spain; 9 Service of Anesthesia, Área Sanitaria de Pontevedra, Pontevedra, Spain; 10 ICU, Hospital Universitario Son Llátzer, Palma, Spain; 11 Faculty of Sports Sciences and Physiotherapy, Universidad Europea de Madrid, Madrid, Spain; 12 ICU, Hospital Universitario de Tarragona Juan XXIII, Tarragona, Spain; 13 Fundación Micellium, La Eliana, Valencia, Spain

**Keywords:** Intraabdominal candidiasis, Echinocandins, Liposomal amphotericin B, PK/PD, Guidelines, Decision algorithm, Intraabdominal penetration, Antifungal stewardship

## Abstract

**Background:**

Regardless of the available antifungals, intraabdominal candidiasis (IAC) mortality continues to be high and represents a challenge for clinicians.

**Main body:**

This opinion paper discusses alternative antifungal options for treating IAC. This clinical entity should be addressed separately from candidemia due to the peculiarity of the required penetration of antifungals into the peritoneal cavity. Intraabdominal concentrations may be further restricted in critically ill patients where pathophysiological facts alter normal drug distribution. Echinocandins are recommended as first-line treatment in guidelines for invasive candidiasis. However, considering published data, our pharmacodynamic analysis suggests the required increase of doses, postulated by some authors, to attain adequate pharmacokinetic (PK) levels in peritoneal fluid. Given the limited evidence in the literature on PK/PD-based treatments of IAC, an algorithm is proposed to guide antifungal treatment. Liposomal amphotericin B is advocated as first-line therapy in patients with sepsis/septic shock presenting candidemia or endophthalmitis, or with prior exposure to echinocandins and/or fluconazole, or with infections by *Candida glabrata*. Other situations and alternatives, such as new compounds or combination therapy, are also analysed.

**Conclusion:**

There is a critical need for more robust clinical trials, studies examining patient heterogeneity and surveillance of antifungal resistance to enhance patient care and optimise treatment outcomes. Such evidence will help refine the existing guidelines and contribute to a more personalised and effective approach to treating this serious medical condition. Meanwhile, it is suggested to broaden the consideration of other options, such as liposomal amphotericin B, as first-line treatment until the results of the fungogram are available and antifungal stewardship could be implemented to prevent the development of resistance.

## Background

Complicated intraabdominal infections (cIAIs) are severe infections, which is challenging for clinicians. An intraabdominal infectious focus could be detected in two-thirds of all surgical patients presenting with sepsis [1], and epidemiological data show that cIAIs are the second most common source in patients where sepsis was the immediate cause of death [2]. cIAIs are generally polymicrobial [3, 4], and treatment is based on effective source control as the main objective to reduce mortality [5] and appropriate therapy. However, regardless of continuous efforts, the mortality of this entity continues to be high, ranging from 20 to 60% [6].

Appropriate therapy should be considered to provide the best antimicrobial efficacy against the infecting pathogens and the greatest minimisation of the possibility of developing resistance. The polymicrobial nature of the indigenous intestinal microbiota complicates this goal of appropriateness; the project AGORA, an international task force from 79 countries, is a good example of the scientific community's efforts to optimise the rational use of antimicrobials for patients with cIAIs [7]. The intestinal tract is an important reservoir for antibiotic-resistant bacteria [7]; a recent study in distinct European regions showed that antimicrobial resistance is common in isolates from critically ill patients with cIAIs [8].

Of importance, the volume of distribution can be significantly increased in the presence of intraabdominal disease [9], especially in critically ill patients where several pharmacokinetic (PK) alterations can lead to drug underdosing [10]. All these could lead to insufficient intraabdominal levels of the drug to attaint values of pharmacokinetic/pharmacodynamic (PK/PD) parameters predicting efficacy and avoiding emergence of resistant variants.

## Intraabdominal candidiasis (IAC)

IAC is caused by the overgrowth of *Candida* species within the abdominal cavity. It primarily affects critically ill patients, those undergoing major abdominal surgeries or individuals with compromised immune systems. Timely and effective treatment is essential to improve patient outcomes and reduce mortality rates. While guidelines exist for the management of IAC [11–15], there remains a crucial need to gather more evidence to support the recommended therapy.

Intensive care unit (ICU) patients are at the highest risk for invasive candidiasis [5]. After ICU admission, the rapid colonisation of mucocutaneous surfaces of patients [16] represents an important risk factor for candidemia [17], an entity with a mortality rate as high as 45% in a recently published multicentre European study [18]. In turn, alterations in the gastrointestinal microbiome precede IAC [19]. *Candida* peritonitis is the predominant invasive candidiasis after candidemia in the ICU [11]. Five species account for 92% of cases of invasive candidiasis: *Candida albicans*, *Candida glabrata*, *Candida tropicalis*, *Candida parapsilosis* and *Candida krusei* [20].

*Candida* spp. are temporary or permanent parts of the normal endogenous flora in the gut in 40–50% of humans [16]. It is normally controlled by nearby bacteria and the host immune system [7]. However, when the gastrointestinal microflora within the host is altered by multiple possible factors (surgery, antibiotic treatments, immunosuppression, etc.), *Candida* invasion and dissemination within the abdominal cavity may occur. IAC is not always accompanied by candidemia; in fact, a recent study reported that only 6.9% patients with IAC had concomitant blood cultures positive for *Candida* spp. [21]. Thus, diagnosis of IAC in the absence of bloodstream infection represents a challenge [22], partly due to the lack of a non-culture-based gold standard method. In addition, when *Candida* is present in intraabdominal samples, the differentiation between contamination, colonisation and infection is not simple since mixed infections with bacteria are frequent [5] and up to 80% of patients with peritonitis are colonised with *Candida* spp. [22].

IAC encompasses complex and highly heterogeneous types of infection [23], with even worse outcomes than bacterial cIAIs [24]. In an observational, multicentre, prospective study in critically ill patients with community-onset IAIs, *Candida* spp. isolation from peritoneal fluid was identified as a risk factor for mortality [25]. Mortality of IAC may exceed 50% [26], regardless that the introduction of echinocandins in the early 2000s was an important advance in managing invasive fungal infections [27]. The occurrence of non-bloodstream invasive candidiasis has increased in the last decade [28], with epidemiological data showing a progressive transition from *C. albicans* to non-*albicans Candida* species as etiological agents [29]. Several *Candida* species (*C. albicans*, *C. auris*, *C. tropicalis*, *C. parapsilosis*) have been deemed critical/high importance to human health and included in the WHO fungal priority pathogens list [30]. But of all of them, C. *auris* is the most worrisome, and it is the first fungal pathogen classified by the CDC as an urgent public threat due to its association with increased mortality, the potential for developing pan-drug resistance and its ability to become entrenched in the hospital environment [31]. In the last 2–3 years, there has been a significant increase in the number of cases caused by *C. auris* in USA [32] with outbreaks also reported in various European countries [33]. This was accompanied by a tripling of the number of cases caused by echinocandin-resistant isolates [31, 34].

Its incidence, the risk of developing resistance in species as *C. auris* and the high mortality associated with IAC make essential to seek strategies to optimise antifungal treatment.

## PK/PD principles for optimisation of IAC treatment

The management of IAC is a complex scenario where conditions other than the antifungal treatment (the patient’s condition, age, infection site, an early and adequate control of the infectious source [11, 20], etc.) are determining factors for the outcome. A recent study analysing the real-life management of this entity showed that not all patients with *Candida* peritonitis received antifungal treatment in real clinical practice [26]. This review does not intend to analyse when antifungal treatment should be initiated based on the risk factors for invasive candidiasis or diagnostic methods, as have been addressed in other published articles [3, 11, 35, 36]. Rather, it aims to focus on, once the decision to initiate an antifungal treatment has been made, what criteria should be followed to ensure the best treatment option.

To maximise antimicrobial efficacy and minimise the emergence of resistance, the treating drug should attain adequate concentrations in the site of infection. This simple concept limits the inference of the information obtained in bloodstream infections to the intraabdominal site. Being the central compartment, the bloodstream represents the easiest site for drug monitoring and PK/PD assessment of drug efficacy. Nevertheless, PK variability is frequent in critically ill patients due to different factors [9, 10]. In the critically ill patient, there is great variability in pharmacokinetic parameters, and there may be an increase in the volume of distribution and renal clearance in the initial phases of the patient with cIAIs. These changes may particularly affect hydrophilic drugs [37] such as beta-lactams and echinocandins. If dosing is not readjusted in the presence of these changes, drug concentrations will not be sufficient to cover pathogens exhibiting less susceptibility to the treating drug, thus favouring the emergence of resistant mutants. To further complicate dosing, in cIAIs there is an impaired tissue penetration which, together with the presence of indwelling surgical drains, may alter drug PK [38]. Other important covariates in altering effective drug concentrations are body weight, serum albumin concentrations and application of extracorporeal treatments [39, 40].

Critical patients with fungal infections are at the highest need for optimal therapy since a deficient state of the immune system is an underlying condition for infection. Thus, in critically ill patients with IAC, where all the above-mentioned risk factors for suboptimal treatment and elevated mortality are frequently present, the main target to ensure an adequate coverage of the infecting species is to attain optimal antifungal concentrations in peritoneal fluid.

## PK/PD analysis of antifungal options for the treatment of IAC

The currently recommended therapy for IAC typically involves antifungal agents, such as fluconazole or echinocandins, administered either intravenously or even intraperitoneally. While these guidelines are based on existing data and expert consensus, the evidence supporting these recommendations remains limited. Clinical trials with a substantial number of participants comparing different antifungal agents, dosing regimens and durations of therapy are scarce.

Conducting large-scale clinical trials for IAC faces several challenges. Firstly, the condition is relatively uncommon, leading to difficulties in enrolling a sufficient number of patients for a robust study. Secondly, patients with IAC often have complex medical conditions, comorbidities and other infections, which make it challenging to isolate the impact of a specific antifungal therapy on outcomes. Additionally, there may be ethical concerns in conducting placebo-controlled trials, as prompt antifungal treatment is vital to prevent disease progression and associated complications. IAC presents with varying degrees of severity, and patient populations may differ significantly in terms of immune status, underlying conditions and comorbidities. The efficacy of a particular antifungal agent may not be consistent across all subgroups, making it essential to explore treatment response in specific patient subsets. Further research is needed to identify predictive factors that may guide treatment selection and optimise therapy for individual patients.

In recent years, the emergence of antifungal resistance has become a growing concern in treating *Candida* infections, including IAC. Monitoring and understanding the patterns of antifungal resistance are critical to ensure the effectiveness of recommended therapies. Large-scale surveillance studies are necessary to assess the prevalence and trends of antifungal resistance among *Candida* isolates from intraabdominal infections.

The financial burden associated with antifungal therapy for IAC should not be overlooked. Novel antifungal agents can be costly, and prolonged therapy may lead to increased healthcare expenses. Comparative effectiveness research is needed to evaluate the cost-effectiveness of different treatment options and provide valuable insights for healthcare policymakers.

Three drug classes, azoles, echinocandins and polyenes, represent the main antifungal armamentarium against *Candida* infections. *C. albicans* continues to be the most frequent species isolated from IACs, but others, some less susceptible to antifungals, begin to gain weight [41, 42]. In the SENTRY antifungal surveillance programme, the frequency of *C. albicans* decreased from 57.4% in 1997–2001 to 46.4% in 2015–2016 [42]. In parallel, this study also reported a gradual and global increase of *C. glabrata* as a causative agent of invasive candidiasis since 1997, with the highest rates of resistance to fluconazole in North America and Asia–Pacific (10.6% and 6.8%, respectively) [42]. The emergence of azole resistance in *Candida* species [43] as well as their interaction with cytochrome P450 leading to drug–drug interactions represents major inconveniences to treatment [44] in critically ill patients.

Echinocandins are concentration-dependent drugs, and their clinical efficacy is related to PK/PD targets as AUC/MIC and Cmax/MIC [37]. The standard dosing regimens are 100 mg/day for micafungin, 200 mg (loading dose) followed by 100 mg/day for anidulafungin and 70 mg (loading dose) followed by 50 mg/day for caspofungin. Table 1 shows serum PK data of the three echinocandins in healthy volunteers and in critically ill patients from a recently published meta-analysis analysing 17 PK studies [37]. According to this article, in critically ill patients the AUC_0-24 h_ was lower than in healthy volunteers for anidulafungin and micafungin, but not for caspofungin [37]. However, controversial results about caspofungin concentrations in critical patients are found in the literature [38, 44, 45]. Echinocandins are highly bound to albumin (> 95%) [37, 46, 47]; thus, considering that only the unbound fraction is active and passively diffuses to the extravascular space, penetration into the peritoneal fluid is highly compromised for these antifungals. Recently, in a population PK model in critically ill patients, several factors with potential impact on micafungin exposure has been proposed, such as increased bodyweight, decreased plasma proteins, higher disease severity score, renal failure and renal replacement therapy, and liver impairment [48].

**Table 1:** Reported pharmacokinetic data in serum for echinocandins in healthy volunteers and critically ill patients

	Healthy volunteers		Critically ill patients	
	Cmax [95%CI]	AUC_0-24 h_ [95%CI]	Cmax [95%CI]	AUC_0-24 h_ [95%CI]
Caspofungin	9.94 [8.99–10.89]	100.47 [87.50–113.44]	8.69 [7.67–9.70]	111.88 [98.44–125.33]
Micafungin	N/A	136.40 [126.73–146.07]	N/A	100.71 [84.59–116.83]
Anidulafungin	7.16 [6.62–7.71]	107.77 [99.72–115.82]	5.75 [5.21–6.29]	89.31 [82.05–96.56]

Cmax (mg/L) and AUC_0-24 h_ data (mg/L × h) for total drug of the three echinocandins in plasma from healthy volunteers and critically ill patients [37]

PK parameters of echinocandins appear to be affected by weight [49]. However, and despite having recommended dose increases for the different echinocandins in these patients, there is insufficient evidence to link this recommendation with better therapeutic results.

Table 2 shows PK data in peritoneal fluid from published studies [38, 39, 50]. The percentage of reduction in peritoneal concentrations of echinocandins with respect to serum concentrations was reported to be approximately 33% [38, 39, 51]. Table 3 shows MIC_90_ values of echinocandins for different *Candida* species [52, 53] and CLSI [54] and EUCAST [55] breakpoints values. Table 4 shows the maximum MIC values which would be covered by concentrations in peritoneal fluid considering the classical target AUC/MIC values defined by Andes et al. (3000 for all *Candida* species, 5000 for non-*C. parapsilosis* species and 285 for *C. parapsilosis*) [56]. It should be noted that these reference values were obtained with PK/PD parameters in blood, and its application to peritoneal concentrations has not been validated.

**Table 2:** Reported pharmacokinetic data in peritoneal fluid for the three echinocandins

	Cmax in PF	AUC_0-24 h_ in PF
Caspofungin [38, 39]	0.5 ± 0.4 1.8 ± 0.9	8.8 ± 7.8 26.0 ± 9.9
Micafungin [39]	0.9 ± 0.7 2.4 ± 1.1	18.8 ± 14.1 44.9 ± 16.3
Anidulafungin [39, 50]	0.9 ± 0.5 2.6 ± 2.2	16.8 ± 8.2 34.4 ± 20.2

Mean ± standard deviation

Pharmacokinetic data (Cmax, mgL; AUC_0-24 h_, mg/L × h) of the three echinocandins in peritoneal fluid (PF) according to published articles (references are shown in parentheses)

**Table 3:** In vitro susceptibility and breakpoints of echinocandins for the main *Candida* species

	Caspofungin [53]			Micafungin [52]			Anidulafungin [52]		
	MIC_90_	CLSI	EUCAST^a^	MIC_90_	CLSI	EUCAST	MIC_90_	CLSI	EUCAST
*C. albicans*	0.03	≥ 1		0.015	≥ 1	> 0.016	0.06	≥ 1	> 0.032
*C. glabrata*	0.06	≥ 0.5		0.03	≥ 0.25	> 0.032	0.12	≥ 0.5	> 0.064
*C. parapsilosis*	0.5	≥ 8		1	≥ 8	> 2	2	≥ 8	> 4
*C. tropicalis*	0.06	≥ 1		0.03	≥ 1	N/A	0.06	≥ 1	> 0.064

^a^Isolates susceptible to anidulafungin and micafungin should be considered susceptible to caspofungin

MIC_90_ values (mg/L) of echinocandins, CLSI [54] and EUCAST [55] breakpoints for different *Candida* species

**Table 4:** PK/PD analysis in plasma and peritoneal fluid for the three echinocandins

	Plasma			Peritoneal fluid		
	Maximum MIC for all *Candida* spp.	Maximum MIC for Non-*C.parapsilosis*	Maximum MIC for *C.parapsilosis*	Maximum MIC for all *Candida* spp.	Maximum MIC for Non-*C. parapsilosis*	Maximum MIC for *C. parapsilosis*
Caspofungin [38, 39]	HV 0.033 CI 0.037	HV 0.020 CI 0.022	HV 0.35 CI 0.39	0.003 -0.009	0.0018- 0.0052	0.03–0.09
Micafungin [39]	HV 0.045 CI 0.034	HV 0.028 CI 0.020	HV 0.48 CI 0.35	0.006–0.015	0.004–0.009	0.066–0.16
Anidulafungin [39, 50]	HV 0.036 CI 0.030	HV 0.022 CI 0.018	HV 0.38 CI 0.31	0.006–0.011	0.0034–0.0069	0.06–0.12

HV: Healthy volunteers; CI: critically ill patients

Maximum MIC values (mg/L) which would be covered by concentrations in peritoneal fluid (see Table 2 for AUC_0-24 h_ values) considering the classical target AUC_0-24 h_/MIC values defined by Andes et al. (3000 for all *Candida* species, 5000 for non-*C. parapsilosis* species and 285 for *C. parapsilosis*) (56)

According to Table 3, except for anidulafungin against *C. albicans* and *C. glabrata*, MIC_90_ values do not exceed breakpoints values; however, they are far from the maximum MIC values covered by concentrations in peritoneal fluid (Table 4). If MIC values in Table 4 are considered, the percentage of isolates from sterile sites covered by anidulafungin would be 10.3% for *C. albicans* and < 1% for other *Candida* species according to the MIC distribution in a recent worldwide surveillance study [41]. Therefore, since drug concentrations are suboptimal in peritoneal fluid, there is a real chance of promoting emergence of antifungal resistance in intraabdominal *Candida*, making of intraabdominal microbiota a reservoir of non-susceptible isolates, as occurs with bacteria. Mean peritoneal concentrations of the three echinocandins were reported to be always below the mutant prevention concentrations in a recent study [39]. In vitro, echinocandin concentrations < 2 mg/L led to selection of resistance mutations in *C. glabrata* isolates [57]. Emergence of resistance was rapid in the laboratory; by exposing *C. glabrata* to a range of growing concentrations of micafungin, echinocandin-resistant mutant colonies were generated in less than 48 h of incubation [58]. For all this, abdominal candidiasis has been pointed out as a hidden reservoir of echinocandin resistance, with 100% therapeutic failures despite source control interventions [59].

Echinocandins at standard doses are recommended at first-line therapy for candidemia in non-neutropenic patients in different clinical guidelines [11–15]. In the guidelines of the Infectious Diseases Society of America, strong recommendation (high-quality evidence) supports both the recommendation of echinocandins, at standard doses, as initial therapy, and of lipid formulation of amphotericin B (3–5 mg/kg daily) in patients with suspected azole- and echinocandin-resistant *Candida* infections [12]. In the ESCMID guidelines, the level of recommendation is “strong” for the use of echinocandins and “moderate” for liposomal amphotericin B [13].

However, there is mounting evidence in the literature, showing that echinocandin exposure is suboptimal in critically ill patients and dose adjustments would be necessary [27, 37, 39, 44, 60–62]. To this end, therapeutic drug monitoring has been postulated as useful tool in patients at risk of suboptimal exposure [27, 44].

Since the introduction of echinocandins in clinical practice at the beginning of this century, reports on development of resistance during or after echinocandin exposure have been found in the literature [63–68]. The cross-resistance to echinocandins and azoles in *C. glabrata* is of high concern [68].

Clinical guidelines abridge practical recommendations to optimise evidence-based treatments. A recent study including 64 centres in 20 European countries has shown that guideline adherence predicts survival in candidemia [18]. However, one of the limitations of guidelines is that recommendations are sometimes too general and could not be adequate for certain circumstances, as for IAC. IAC represents a frequent entity among critically ill patients, with characteristics in relation to dosages and drug distribution, and it is not addressed in the clinical guidelines issued by the principal infectious diseases’ scientific societies, probably due to the lack of good quality treatment evidence for this entity [11, 69]. The current guidelines assume the same criteria as for candidemia [12], without any other type of assessment, regardless of published data on the low peritoneal drug penetration [38, 39, 50, 51].

## Analysis of potential alternatives

Two important facts should make us move forward in response to the challenge that represents with a growing trend, invasive fungal infections: first, the unacceptable high mortality rate despite the compounds available as treatment; and second, the need to preserve the antifungal armamentarium, especially limited compared to the antibacterial one. Lessons learned from the emergence of bacterial resistance due to the incorrect use of antibiotics should guide us to optimise using antifungal compounds. It is a reality that the use of antifungals has increased in recent decades and with it the number of resistant strains [42, 60]. Likewise, there is a trend towards greater isolation of species that are resistant to older antifungals, indicating that non-*albicans* species find a growing niche [42, 70]. Although far from being common, multidrug-resistant *C. auris* isolates are increasingly detected worldwide [71]. To avoid worrisome scenarios and to preserve current antifungals until new compounds are available, three actions could be considered.

### Reassessment of echinocandin doses [60]

In parallel with an increasing number of articles informing on suboptimal drug exposure in critically ill patients, there is an increasing request on the need to update recommended echinocandin dosages in clinical guidelines [60, 72]. Echinocandins are well-tolerated antifungals since they inhibit beta-glucan synthesis, a target not found in humans [27, 44, 73]. A Monte Carlo simulation justified higher doses of echinocandins, especially in those patients weighing > 70 kg and infected by non-*albicans* species [51]. Several clinical trials have investigated high dosages, with a favourable safety outcome [74–77]. However, no significant differences in efficacity were found when compared with standard doses in clinical trials [76, 77], a fact which could be related to the well-described limitation of this type of studies to include enough patients infected by isolates exhibiting high MIC values (patients who can make the difference in efficacy between the two treatments) due to the limited sample size [60]. In the absence of clinical data, the prudent setting of cut-off values exclusively guided by PK/PD principles would be an option to be considered. Nevertheless, research should be conducted to determine whether humans may experience issues associated with high dosing, as reported in a mouse model in which an initial decrease in *C. glabrata* in the gut was followed by a rebound to original levels now characterised by a high level of resistant yeast [78]. Rezafungin is a next generation echinocandin derived from anidulafungin. Its main advantages are linked to its pharmacokinetic characteristics that allow once-a-week administration, enhance its penetration to difficult-to-reach anatomical sites such as the peritoneal cavity and lower the probability of resistance promotion [79]. However, we need more clinical studies on the performance of rezafungin in patients with cIAIs. In development, fosmanogepix is a new potential alternative targeting the fungal enzyme Gwt1 and exhibiting a high oral bioavailability that has shown promising efficacy and safety results in an open-label Phase II study [80]. Once more, it would be desirable, as for every new compound, to be tested in the treatment of IAC during the clinical development stage.

### Is there enough evidence to consider polyenes as first-line therapeutic alternative?

Amphotericin B is a well-known drug as it was discovered more than 70 years ago. It has shown to be a nephrotoxic drug and it is placed as second-line antifungal in most guidelines. Some confusion arises with this side effect between its formulations which have similar clinical and microbiological efficacy but differences in toxicity. Keane et al. conducted a systematic review of a head-to-head comparison of amphotericin against other antifungals, and there was no evidence of clinical inferiority [68]. Liposomal amphotericin B is a lipid-based formulation of amphotericin B reducing the risk of nephrotoxicity of amphotericin deoxycholate [81–83]. It also allows considerable dose increases with respect to the conventional formulation, which contributes to improving the antifungal effectivity [84]. The literature shows that fewer severe drug-related adverse events occur with the liposomal formulation than with the conventional one [44, 82]. In two recent studies evaluating liposomal amphotericin B as outpatient therapy [85, 86], although approximately 50% of patients presented some renal injury during treatment, adverse events could be well managed, and only 12% of patients required readmission for these events [85] or treatment discontinuation in only one patient [86]. High doses of the drug were identified as a risk factor for nephrotoxicity [85]. On the other hand, a favourable safety aspect of liposomal amphotericin B is that drug–drug interactions are irrelevant [87], an important factor in critically ill patients.

Liposomal amphotericin B presents good antimicrobial efficacy against *Candida* spp. and a very low risk of development of resistance [87, 88]. It displays concentration-dependent fungicidal activity with a prolonged post-antifungal effect in time-kill studies [82]. The Cmax/MIC ratio seems the target PK/PD parameter linked to efficacy, although more information is required [82, 89]. More data on the pharmacokinetics of liposomal amphotericin B in critically ill patients would also be desirable [44]. A recently published study showed considerable intra- and inter-patient variability for plasma Cmax and AUC, without identified responsible covariates [89]. In that study, Cmax (mg/L) was 20.0 [14.1–27.9] and 43.7 [41.3–64.4] with the 3 mg/kg/day and 5 mg/kg/day doses, respectively [89]. Of maximum interest was that the values of these PK parameters, which were measured in critically ill patients, were not significantly different from those in healthy volunteers [89]. Since PK/PD targets have only been poorly defined for liposomal amphotericin B, the added value of calculating Cmax/MIC or AUC/MIC target attainment is unclear. Hence, the potential clinical consequences of the large variability in exposure cannot be derived [89]. In addition, due to the lipophilic characteristics of amphotericin B, the exposure in different tissues might differ, and it might be less affected by pathophysiological changes than hydrophilic drugs [89, 90]. Furthermore, the authors have measured total amphotericin B concentrations while the active amphotericin B is in the liposome [88, 91]. Of importance in critically ill patients, neither dialysis nor hemofiltration reduced Cmax or AUC values of amphotericin B in serum [84].

Even fewer data on concentrations in peritoneal fluid could be found in the literature. For amphotericin desoxycholate, some years ago a good linear correlation was found between serum and peritoneal levels [92], but no data are available for liposomal amphotericin B. A published case series in paediatric patients concluded that peritoneal liposomal amphotericin B concentrations were significantly lower than in plasma, hampering to attain the Cmax/MIC target value [93]. Considering MIC_90_ values for the different *Candida* species (MICs of 0.5–1 mg/L) [41, 52, 53], Cmax of liposomal amphotericin B in peritoneum should be at least 4.5 mg/L. To our knowledge, no more data on peritoneum concentrations are available, making difficult its PK/PD assessment for IAC. The increase in vascular permeability due to the destruction of infected tissues has been postulated as the fact increasing the transfer of liposomal amphotericin B to infected regions resulting in higher antifungal concentrations and in vivo antifungal effects [94]. In one case, a much higher concentration of amphotericin B was observed in the infected lung lesion than in uninfected lung tissue which support the hypothesis that liposomal amphotericin B accumulates in lesions of fungal infection [88, 91, 95]. In the peritoneal study, half of the patients received liposomal amphotericin B as prophylaxis [93], making it difficult to draw conclusions. According to the literature, liposomal amphotericin B attains fungicidal activity concentrations in difficult-to-reach compartments, as peritoneum, among others [68]. A systematic review concluded that no differences in clinical efficacy could be found between amphotericin B, echinocandins or voriconazole in critically ill patients with invasive candidiasis [68]. For this reason, the authors suggested that all three types of antifungals should be considered first-line therapy, and the guide for the definitive choice should be local *Candida* species epidemiology and susceptibility [68]. This recommendation of advancing liposomal amphotericin B into first-line therapy in updated guidelines are supported by other authors [69, 96, 97] based on its low propensity to elicit acquired resistance [88] and especially in cases of previous azole exposure [97]. Although fluconazole could not be considered as appropriate empirical therapy for invasive candidiasis due to the existing resistance in non-albicans species [68], further de-escalation to fluconazole reaches consensus in case of susceptibility of the *Candida* isolate when the patient is clinically stable [11].

An adding effect of liposomal amphotericin B is the powerful action against biofilm formation. Peritoneal biofilm formation can alter MIC and minimum bactericidal concentrations (MBC) due to the growth of matrix-enclosed bacteria. Experimental studies have described the presence and evolution of bacterial biofilms (mature multilayer polymicrobial biofilms) on the peritoneal surface during severe secondary peritonitis, with deep penetration in the abdominal wall after 48–72 h of puncture/ligation [98]. Fungal superinfection of such cavities is a source of non-adequate antifungal penetration.

### Consideration of combination therapy followed by de-escalation when microbiological information is available

Combination of antifungals seems attractive for some fungal infections with adequate resolution. The main concern is to determine whether a combination of antifungal drugs could develop antagonism or deleterious effects from the individual drugs that have been administered.

On the other hand, combination of antifungals has been widely addressed to maximise the antifungal effect through the potential synergistic effect of different compounds [99]. Although in vitro and in vivo (animal models) results showed promising [100–104], there is limited clinical evidence for a single combination. A systematic review identified 92 studies on combination antifungal therapy in the literature, 55 of them referring to clinical practice [104]. Combinations included azoles plus echinocandins (36%), 5-flucytosine combination therapies (24%), polyenes plus azoles (18%), polyenes plus echinocandins (16%) and other types of combination therapy (6%) [104]. Targets were “difficult-to-treat infections (endocarditis, osteoarticular, etc.)” or “difficult-to-treat infecting *Candida* species” [104]. Only one study addressed fungal peritonitis with combination therapy (intravenous amphotericin B and oral flucytosine with deferred catheter replacement) and showed a lower technique failure rate but similar length of hospitalisation and mortality [105]. Due to the high heterogeneity of data from studies included in the review, specific conclusions could not be drawn as a basis for their practical clinical application. In view of the growing number of fungal infections, their high mortality and the limited number of antifungals available, further research on combined therapies in clinical trials with an adequate sample size of patients suffering from pathologies such as IAC, which represents a challenge not only for clinical efficacy but also to preserve the susceptibility of intestinal microbiota, would be welcome.

**Fig. 1:**
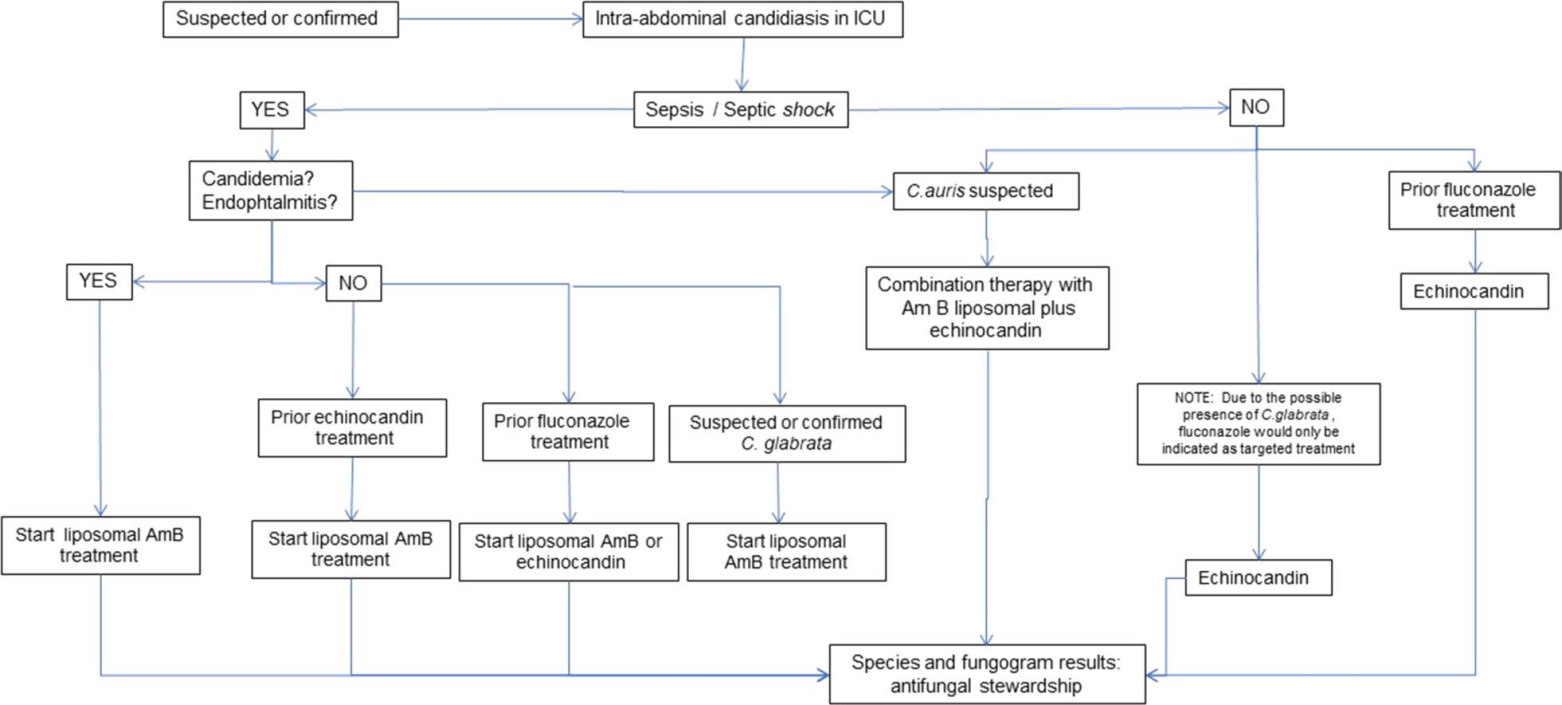
Proposed algorithm on the use of antifungals in ICU patients with intraabdominal candidiasis

Considering all the arguments mentioned above, the authors propose the algorithm in Fig. 1 on the use of antifungals in ICU patients with intraabdominal candidiasis. By analysing this algorithm, basic recommendations could be expressed as:

Step one: If sepsis or septic shock with candidemia or endophthalmitis is present, because echinocandins do not reach therapeutic concentrations in eyes and the central nervous system [106, 107], liposomal amphotericin B should be the option.

Step two: In the case of sepsis or septic shock without the two above-mentioned sources of infection, prior exposure to antifungals will determine the choice of the drug. If previous treatment with echinocandins and/or fluconazole, liposomal amphotericin B is preferred, in the absence of previous treatment, echinocandins or liposomal amphotericin B is recommended. If *C. glabrata* is highly suspected, liposomal amphotericin B should be initiated based on reports of treatment failure with echinocandins due to resistance [108, 109].

Step three: If there is an epidemiological risk of *C. auris*, a combined liposomal amphotericin B+ echinocandin treatment is recommended due to the high resistance of the fungus.

Step four: If sepsis/shock is not present, treatment may be more conservative. We suggest starting an echinocandin in case of previous fluconazole treatment or suspected *C. glabrata* infection. Although echinocandin resistance may be present in *C. glabrata*, given the stability of the patient, treatment can be adjusted with the fungogram at 48 h. If the fungogram is not available, liposomal amphotericin B is recommended until species identification.

Finally, antifungal stewardship is always recommended once the fungogram results are available.

## Conclusions

According to the literature, the percentage of reduction in echinocandin concentrations in peritoneum with respect to serum is approximately 33% [38, 39, 51]. Our PK/PD analysis of published data showed that MIC_90_ values of echinocandins are lower than breakpoints values except for anidulafungin against *C. albicans* and *C. glabrata*. However, these MIC_90_ are far above the maximum MIC values covered by concentrations in peritoneal fluid. This implies that the percentage of isolates from sterile sites covered by anidulafungin would be < 10% considering the MIC distribution in a recent worldwide surveillance study [41]. Suboptimal drug concentrations in peritoneal fluid represent a risk for emergence of antifungal resistance in intraabdominal *Candida*, making of intraabdominal microbiota a reservoir of non-susceptible isolates, as occurs with bacteria. Further studies determining concentrations of antifungals in peritoneal samples as the one planned [110] or the recently published with voriconazole [111] represent the first step to achieve the adequate dosing in this entity. A current opinion in the literature indicates that guidelines should be updated by separating the recommendations for candidemia and abdominal candidiasis with a revision of echinocandin doses and the inclusion of other antifungals such as liposomal amphotericin B. In the meantime, an algorithm is proposed considering, in addition to echinocandins, liposomal amphotericin B as first-line therapy facing sepsis and candidemia or prior antifungal treatment. To address *C. auris*, a combination therapy of liposomal amphotericin B and echinocandin is proposed. Once species and fungogram are known, antifungal stewardship is warranted to preserve as much as possible available antifungals.

In conclusion, while current guidelines provide valuable recommendations for the management of IAC, the evidence supporting these therapeutic approaches remains limited. To enhance patient care and optimise treatment outcomes, there is a critical need for more robust clinical trials, studies examining patient heterogeneity and surveillance of antifungal resistance. Such evidence will help refine the existing guidelines and contribute to a more personalised and effective approach in treating this serious medical condition. Investing in research and fostering collaborations among healthcare professionals and researchers can improve our understanding of IAC and advance the field of antifungal therapy for the benefit of patients worldwide.

## Data Availability

Not applicable.

## Abbreviations

AUC: Area under the curve of drug concentrations versus time
CLSI: Clinical & Laboratory Standards Institute
Cmax: Maximum concentration
EUCAST: European Committee on Antimicrobial Susceptibility Testing
IAC: Intraabdominal candidiasis
ICU: Intensive care unit
cIAIs: Complicated intraabdominal infections
MIC: Minimum inhibitory concentration
MBC: Minimum bactericidal concentrations
PK: Pharmacokinetic
PK/PD: Pharmacokinetic/pharmacodynamic

## References

[1] Hecker A, Reichert M, Reuß CJ, Schmoch T, Riedel JG, Schneck E (2019). Intra-abdominal sepsis: new definitions and current clinical standards. Langenbecks Arch Surg.

[2] Rhee C, Jones TM, Hamad Y, Pande A, Varon J, O’Brien C (2019). Prevalence, underlying causes, and preventability of sepsis-associated mortality in US acute care hospitals. JAMA Netw Open.

[3] Mazuski JE, Tessier JM, May AK, Sawyer RG, Nadler EP, Rosengart MR (2017). The surgical infection society revised guidelines on the management of intra-abdominal infection. Surg Infect (Larchmt).

[4] Shah PM, Edwards BL, Dietch ZC, Guidry CA, Davies SW, Hennessy SA (2016). Do polymicrobial intra-abdominal infections have worse outcomes than monomicrobial intra-abdominal infections?. Surg Infect (Larchmt).

[5] Lagunes L, Rey-Pérez A (2019). What´s new in intraabdominal candidiasis in critically ill patients, a review. Hosp Pract (1995).

[6] van Ruler O, Boermeester MA (2017). Surgical treatment of secondary peritonitis. Chirurg.

[7] Sartelli M, Weber DG, Ruppé E, Bassetti M, Wright BJ, Ansaloni L (2016). Antimicrobials: a global alliance for optimizing their rational use in intra-abdominal infections (AGORA). World J Emerg Surg.

[8] Vogelaers D, Blot S, van den Berge A, Montravers P, Francois G, Labeau S (2021). Antimicrobial lessons from a large observational cohort on intra-abdominal infections in intensive care units. Drugs.

[9] Adnan S, Paterson DL, Lipman J, Kumar S, Li J, Rudd M (2012). Pharmacokinetics of beta-lactam antibiotics in patients with intra-abdominal disease: a structured review. Surg Infect (Larchmt).

[10] Leon L, Guerci P, Pape E, Thilly N, Luc A, Germain A (2020). Serum and peritoneal exudate concentrations after high doses of β-lactams in critically ill patients with severe intra-abdominal infections: an observational prospective study. J Antimicrob Chemother.

[11] Pemán J, Aguilar G, Valía JC, Salavert M, Navarro D, Zaragoza R (2017). Jávea consensus guidelines for the treatment of Candida peritonitis and other intra-abdominal fungal infections in non-neutropenic critically ill adult patients. Rev Iberoam Micol.

[12] Pappas PG, Kauffman CA, Andes DR, Clancy CJ, Marr KA, Ostrosky-Zeichner L (2016). Clinical practice guideline for the management of candidiasis: 2016 Update by the Infectious Diseases Society of America. Clin Infect Dis.

[13] Cornely OA, Bassetti M, Calandra T, Garbino J, Kullberg BJ, Lortholary O (2012). ESCMID guideline for the diagnosis and management of Candida diseases 2012: non-neutropenic adult patients. Clin Microbiol Infect.

[14] Scudeller L, Viscoli C, Menichetti F, del Bono V, Cristini F, Tascini C (2014). An Italian consensus for invasive candidiasis management (ITALIC). Infection.

[15] Chen SC, Sorrell TC, Chang CC, Paige EK, Bryant PA, Slavin MA (2014). Consensus guidelines for the treatment of yeast infections in the haematology, oncology and intensive care setting, 2014. Intern Med J.

[16] Eggimann P, Garbino J, Pittet D (2003). Epidemiology of Candida species infections in critically ill non-immunosuppressed patients. Lancet Infect Dis.

[17] Alenazy H, Alghamdi A, Pinto R, Daneman N (2021). Candida colonization as a predictor of invasive candidiasis in non-neutropenic ICU patients with sepsis: a systematic review and meta-analysis. Int J Infect Dis.

[18] Hoenigl M, Salmanton-García J, Egger M, Gangneux JP, Bicanic T, Arikan-Akdagli S (2023). Guideline adherence and survival of patients with candidaemia in Europe: results from the ECMM Candida III multinational European observational cohort study. Lancet Infect Dis.

[19] Choy A, Freedberg DE (2020). Impact of microbiome-based interventions on gastrointestinal pathogen colonization in the intensive care unit. Therap Adv Gastroenterol.

[20] Bassetti M, Righi E, Ansaldi F, Merelli M, Scarparo C, Antonelli M (2015). A multicenter multinational study of abdominal candidiasis: epidemiology, outcomes and predictors of mortality. Intensive Care Med.

[21] Bassetti M, Vena A, Giacobbe DR, Trucchi C, Ansaldi F, Antonelli M (2022). Risk Factors for intra-abdominal candidiasis in intensive care units: Results from EUCANDICU Study. Infect Dis Ther.

[22] Fortún J, Buitrago MJ, Gioia F, Gómez-G de la Pedrosa E, Alvarez ME, Martín-Dávila P (2020). Roles of the multiplex real-time PCR assay and β-D-glucan in a high-risk population for intra-abdominal candidiasis (IAC). Med Mycol.

[23] Vergidis P, Clancy CJ, Shields RK, Park SY, Wildfeuer BN, Simmons RL (2016). Intra-abdominal candidiasis: The importance of early source control and antifungal treatment. PLoS ONE.

[24] Esher SK, Fidel PL, Noverr MC (2019). Candida/Staphylococcal polymicrobial intra-abdominal infection: pathogenesis and perspectives for a novel form of trained innate immunity. J Fungi (Basel).

[25] Maseda E, Ramírez S, Picatto P, Peláez-Peláez E, García-Bernedo C, Ojeda-Betancur N (2019). Critically ill patients with community-onset intraabdominal infections: Influence of healthcare exposure on resistance rates and mortality. PLoS ONE.

[26] Dubler S, Laun M, Koch C, Hecker A, Weiterer S, Siegler BH (2017). The impact of real life treatment strategies for *Candida* peritonitis-A retrospective analysis. Mycoses.

[27] Kim HY, Baldelli S, Märtson AG, Stocker S, Alffenaar JW, Cattaneo D (2022). Therapeutic drug monitoring of the echinocandin antifungal agents: Is there a role in clinical practice? A position statement of the Anti-Infective Drugs Committee of the International Association of Therapeutic Drug Monitoring and Clinical Toxicology. Ther Drug Monit.

[28] Ricotta EE, Lai YL, Babiker A, Strich JR, Kadri SS, Lionakis MS (2021). Invasive candidiasis species distribution and trends, United States, 2009–2017. J Infect Dis.

[29] Lamoth F, Lockhart SR, Berkow EL, Calandra T (2018). Changes in the epidemiological landscape of invasive candidiasis. J Antimicrob Chemother.

[30] Fisher MC, Denning DW (2023). The WHO fungal priority pathogens list as a game-changer. Nat Rev Microbiol.

[31] Lyman M, Forsberg K, Sexton DJ, Chow NA, Lockhart SR, Jackson BR (2023). Worsening spread of *Candida auris* in the United States, 2019 to 2021. Ann Intern Med.

[32] Benedict K, Forsberg K, Gold JAW, Baggs J, Lyman M (2023). *Candida auris* -associated hospitalizations, United States, 2017–2022. Emerg Infect Dis.

[33] Kohlenberg A, Monnet DL, Plachouras D (2022). Increasing number of cases and outbreaks caused by *Candida auris* in the EU/EEA, 2020 to 2021. Eurosurveillance.

[34] Centers for Disease Control and Prevention. Increasing Threat of Spread of Antimicrobial-resistant Fungus in Healthcare Facilities. CDC Online Newsroom. https://www.cdc.gov/media/releases/2023/p0320-cauris.html. Accessed 23 August 2023.

[35] Berg DM, Slish JC, Wright M, Gandhi AD, Gandhi MA. Current utilization of antifungal agents for intra-abdominal infections categorized by patient risk factors during surgical procedures: A literature review. J Pharm Pract. 2022;089719002211087. 10.1177/08971900221108716 35705106

[36] Martin-Loeches I, Antonelli M, Cuenca-Estrella M, Dimopoulos G, Einav S, De Waele JJ (2019). ESICM/ESCMID task force on practical management of invasive candidiasis in critically ill patients. Intensive Care Med.

[37] Liu X, Liu D, Pan Y, Li Y (2020). Pharmacokinetic/pharmacodynamics variability of echinocandins in critically ill patients: a systematic review and meta-analysis. J Clin Pharm Ther.

[38] Garbez N, Mbatchi LC, Wallis SC, Muller L, Lipman J, Roberts JA (2022). Caspofungin population pharmacokinetic analysis in plasma and peritoneal fluid in septic patients with intra-abdominal infections: A prospective cohort study. Clin Pharmacokinet.

[39] Gioia F, Gomez-Lopez A, Alvarez ME, Gomez-García de la Pedrosa E, Martín-Davila P, Cuenca-Estrella M (2020). Pharmacokinetics of echinocandins in suspected *Candida* peritonitis: A potential risk for resistance. Int J Infect Dis.

[40] Varghese JM, Roberts JA, Lipman J (2010). Pharmacokinetics and pharmacodynamics in critically ill patients. Curr Opin Anaesthesiol.

[41] Jean SS, Yang HJ, Hsieh PC, Huang YT, Ko WC, Hsueh PR (2022). *In vitro* susceptibilities of worldwide isolates of intrapulmonary *Aspergillus* species and important *Candida* species in sterile body sites against important antifungals: Data from the Antimicrobial Testing Leadership and Surveillance Program, 2017–2020. Microbiol Spectr.

[42] Pfaller MA, Diekema DJ, Turnidge JD, Castanheira M, Jones RN (2019). Twenty years of the SENTRY Antifungal Surveillance Program: results for *Candida* species from 1997–2016. Open Forum Infect Dis.

[43] Branco J, Miranda IM, Rodrigues AG (2023). *Candida parapsilosis* virulence and antifungal resistance mechanisms: a comprehensive review of key determinants. J Fungi (Basel).

[44] Baracaldo-Santamaría D, Cala-Garcia JD, Medina-Rincón GJ, Rojas-Rodriguez LC, Calderon-Ospina CA (2022). Therapeutic drug monitoring of antifungal agents in critically ill patients: Is there a need for dose optimisation?. Antibiotics (Basel).

[45] van der Elst KCM, Veringa A, Zijlstra JG, Beishuizen A, Klont R, Brummelhuis-Visser P (2017). Low caspofungin exposure in patients in intensive care units. Antimicrob Agents Chemother.

[46] Bellmann R, Smuszkiewicz P (2017). Pharmacokinetics of antifungal drugs: practical implications for optimized treatment of patients. Infection.

[47] Yamasaki K, Sakurama K, Nishi K, Tsukigawa K, Seo H, Otagiri M (2022). An in-vitro comparative study of the binding of caspofungin and micafungin to plasma proteins. J Pharm Pharmacol.

[48] Boonstra JM, van der Elst KC, Zijlstra JG, van der Werf TS, Alffenaar JWC, Touw DJ (2022). Population pharmacokinetic model and optimal sampling strategies for micafungin in critically ill patients diagnosed with invasive candidiasis. Antimicrob Agents Chemother.

[49] Payne KD, Hall RG (2016). Dosing of antifungal agents in obese people. Expert Rev Anti Infect Ther.

[50] Pérez Civantos DV, Robles Marcos M, Azanza Perea JR, Pazos Pacheco C, García-Montoto Pérez F, Jerez G-C (2019). Pharmacokinetics of anidulafungin in critically ill patients with *Candida* peritonitis. Int J Infect Dis.

[51] Garbez N, Mbatchi L, Wallis SC, Muller L, Lipman J, Roberts JA (2021). Prospective cohort study of micafungin population pharmacokinetic analysis in plasma and peritoneal fluid in septic patients with intra-abdominal infections. Antimicrob Agents Chemother.

[52] Pfaller MA, Huband MD, Rhomberg PR, Bien PA, Castanheira M (2022). Activities of manogepix and comparators against 1435 recent fungal isolates collected during an International Surveillance Program (2020). Antimicrob Agents Chemother.

[53] Pfaller MA, Carvalhaes C, Messer SA, Rhomberg PR, Castanheira M (2020). Activity of a long-acting echinocandin, rezafungin, and comparator antifungal agents tested against contemporary invasive fungal isolates (SENTRY Program, 2016 to 2018). Antimicrob Agents Chemother.

[54] Clinical and Laboratory Standards Institute (CLSI). Performance Standards for Antifungal Susceptibility Testing of Yeasts, 3rd ed. CLSI supplement M27M44S. Clinical and Laboratory Standards Institute, USA 2022.

[55] European Society of Clinical Microbiology and Infectious Diseases. European Committee on Antimicrobial Susceptibility Testing. Breakpoint tables for interpretation of MICs for antifungal agents, version 10.0, 2020. https://www.eucast.org/fileadmin/src/media/PDFs/EUCAST_files/AFST/Clinical_breakpoints/AFST_BP_v10.0_200204_updatd_links_200924.pdf. Accessed 23 August 2023.

[56] Andes D, Ambrose PG, Hammel JP, van Wart SA, Iyer V, Reynolds DK (2011). Use of pharmacokinetic-pharmacodynamic analyses to optimize therapy with the systemic antifungal micafungin for invasive candidiasis or candidemia. Antimicrob Agents Chemother.

[57] Bordallo-Cardona MA, Marcos-Zambrano LJ, Sánchez-Carrillo C, de la Pedrosa EGG, Cantón R, Bouza E (2018). Mutant prevention concentration and mutant selection window of micafungin and anidulafungin in clinical *Candida glabrata* isolates. Antimicrob Agents Chemother.

[58] Rivero-Menendez O, Navarro-Rodriguez P, Bernal-Martinez L, Martin-Cano G, Lopez-Perez L, Sanchez-Romero I (2019). Clinical and laboratory development of echinocandin resistance in *Candida glabrata*: Molecular characterization. Front Microbiol.

[59] Shields RK, Nguyen MH, Press EG, Clancy CJ (2014). Abdominal candidiasis is a hidden reservoir of echinocandin resistance. Antimicrob Agents Chemother.

[60] Bader JC, Bhavnani SM, Andes DR, Ambrose PG (2018). We can do better: a fresh look at echinocandin dosing. J Antimicrob Chemother.

[61] Xie J, Yang Q, Han X, Dong Y, Zhang T, Li Y (2022). Pharmacokinetic/Pharmacodynamic target attainment of different antifungal agents in de-escalation treatment in critically ill patients: a step toward dose optimization using Monte Carlo simulation. Antimicrob Agents Chemother.

[62] Maseda E, Grau S, Luque S, Castillo-Mafla MP, Suárez-de-la-Rica A, Montero-Feijoo A (2018). Population pharmacokinetics/pharmacodynamics of micafungin against *Candida* species in obese, critically ill, and morbidly obese critically ill patients. Crit Care.

[63] Dannaoui E, Desnos-Ollivier M, Garcia-Hermoso D, Grenouillet F, Cassaing S, Baixench MT (2012). *Candida* spp. with acquired echinocandin resistance, France, 2004–20101. Emerg Infect Dis.

[64] Forastiero A, Garcia-Gil V, Rivero-Menendez O, Garcia-Rubio R, Monteiro MC, Alastruey-Izquierdo A (2015). Rapid development of *Candida krusei* echinocandin resistance during caspofungin therapy. Antimicrob Agents Chemother.

[65] Pfeiffer CD, Garcia-Effron G, Zaas AK, Perfect JR, Perlin DS, Alexander BD (2010). Breakthrough invasive candidiasis in patients on micafungin. J Clin Microbiol.

[66] Ruggero MA, Topal JE (2014). Development of echinocandin-resistant *Candida albicans* candidemia following brief prophylactic exposure to micafungin therapy. Transpl Infect Dis.

[67] Sfeir MM, Jiménez-Ortigosa C, Gamaletsou MN, Schuetz AN, Soave R, van Besien K (2020). Breakthrough bloodstream infections caused by echinocandin-resistant *Candida tropicalis*: an emerging threat to immunocompromised patients with hematological malignancies. J Fungi (Basel).

[68] Keane S, Geoghegan P, Povoa P, Nseir S, Rodriguez A, Martin-Loeches I (2018). Systematic review on the first line treatment of amphotericin B in critically ill adults with candidemia or invasive candidiasis. Expert Rev Anti Infect Ther.

[69] Zaragoza R, Maseda E, Pemán J (2021). Individualized antifungal therapy in critically ill patients with invasive fungal infection. Rev Iberoam Micol.

[70] Bohner F, Papp C, Gácser A (2022). The effect of antifungal resistance development on the virulence of *Candida* species. FEMS Yeast Res.

[71] Geremia N, Brugnaro P, Solinas M, Scarparo C, Panese S (2023). *Candida auris* as an emergent public health problem: a current update on European outbreaks and cases. Healthcare.

[72] Peçanha-Pietrobom PM, Colombo AL (2020). Mind the gaps: Challenges in the clinical management of invasive candidiasis in critically ill patients. Curr Opin Infect Dis.

[73] Yang YL, Xiang ZJ, Yang JH, Wang WJ, Xu ZC, Xiang RL (2021). Adverse effects associated with currently commonly used antifungal agents: A network meta-analysis and systematic review. Front Pharmacol.

[74] Brüggemann RJM, van der Velden WJFM, Knibbe CAJ, Colbers A, Hol S, Burger DM (2015). A rationale for reduced-frequency dosing of anidulafungin for antifungal prophylaxis in immunocompromised patients. J Antimicrob Chemother.

[75] Neofytos D, Huang YT, Cheng K, Cohen N, Perales MA, Barker J (2015). Safety and efficacy of intermittent intravenous administration of high-dose micafungin. Clin Infect Dis.

[76] Betts RF, Nucci M, Talwar D, Gareca M, Queiroz-Telles F, Bedimo RJ (2009). A multicenter, double-blind trial of a high-dose caspofungin treatment regimen versus a standard caspofungin treatment regimen for adult patients with invasive candidiasis. Clin Infect Dis.

[77] Grant VC, Nguyen K, Rodriguez S, Zhou AY, Abdul-Mutakabbir JC, Tan KK (2022). Characterizing safety and clinical outcomes associated with high-dose micafungin utilization in patients with proven invasive candidiasis. Trop Med Infect Dis.

[78] Healey KR, Nagasaki Y, Zimmerman M, Kordalewska M, Park S, Zhao Y (2017). The gastrointestinal tract is a major source of echinocandin drug resistance in a murine model of *Candida glabrata* colonization and systemic dissemination. Antimicrob Agents Chemother.

[79] Guinea J (2023). Rezafungin and invasive candida infections: a new game changing antifungal?. The Lancet.

[80] Shaw KJ, Ibrahim AS (2020). Fosmanogepix: a review of the first-in-class broad spectrum agent for the treatment of invasive fungal infections. J Fungi (Basel).

[81] Walsh TJ, Finberg RW, Arndt C, Hiemenz J, Schwartz C, Bodensteiner D (1999). Liposomal amphotericin B for empirical therapy in patients with persistent fever and neutropenia. N Engl J Med.

[82] Groll AH, Rijnders BJA, Walsh TJ, Adler-Moore J, Lewis RE, Brüggemann RJM (2019). Clinical pharmacokinetics, pharmacodynamics, safety and efficacy of liposomal amphotericin B. Clin Infect Dis.

[83] Botero Aguirre JP, Restrepo Hamid AM. Amphotericin B deoxycholate versus liposomal amphotericin B: effects on kidney function. Cochrane Database Syst Rev. 2015;11: CD010481 10.1002/14651858.CD010481.pub2 PMC10542271 26595825

[84] Heinemann V, Bosse D, Jehn U, Kähny B, Wachholz K, Debus A (1997). Pharmacokinetics of liposomal amphotericin B (Ambisome) in critically ill patients. Antimicrob Agents Chemother.

[85] Burnett YJ, Spec A, Ahmed MM, Powderly WG, Hamad Y (2021). Experience with liposomal amphotericin B in outpatient parenteral antimicrobial therapy. Antimicrob Agents Chemother.

[86] van de Peppel RJ, Schauwvlieghe A, Van Daele R, Spriet I, van’t Wout JW, Brüggemann RJ, et al. Outpatient parenteral antifungal therapy (OPAT) for invasive fungal infections with intermittent dosing of liposomal amphotericin B. Med Mycol. 2020;58(7):874–80. 10.1093/mmy/myz134 PMC7527269 31965178

[87] Maertens J, Pagano L, Azoulay E, Warris A. Liposomal amphotericin B—the present. J Antimicrob Chemother. 2022;77(Suppl_2):ii11–ii20. 10.1093/jac/dkac352 PMC9693760 36426672

[88] Brüggemann RJ, Jensen GM, Lass-Flörl C. Liposomal amphotericin B-the past. J Antimicrob Chemother. 2022;77(2):ii3–ii10. 10.1093/jac/dkac351 PMC9693798 36426673

[89] Van Daele R, Wauters J, Elkayal O, Dreesen E, Debaveye Y, Lagrou K, et al. Liposomal amphotericin B exposure in critically ill patients: a prospective pharmacokinetic study. Med Mycol. 2022;60(10):myac074. 10.1093/mmy/myac074 36124725

[90] Jamal JA, Roger C, Roberts JA (2018). Understanding the impact of pathophysiological alterations during critical illness on drug pharmacokinetics. Anaesth Crit Care Pain Med.

[91] Azanza Perea JR, Barberán J (2012). Liposomal amphotericin B: A unique pharmacokinetic profile. An unfinished story. Rev Esp Quimioter.

[92] van der Voort PHJ, Boerma EC, Yska JP (2007). Serum and intraperitoneal levels of amphotericin B and flucytosine during intravenous treatment of critically ill patients with *Candida* peritonitis. J Antimicrob Chemother.

[93] Tortora F, Dei Giudici L, Simeoli R, Chiusolo F, Cairoli S, Bernaschi P (2022). therapeutic drug monitoring of amphotericin-b in plasma and peritoneal fluid of pediatric patients after liver transplantation: a case series. Antibiotics.

[94] Takemoto K, Kanazawa K (2017). Am Bisome: relationship between the pharmacokinetic characteristics acquired by liposomal formulation and safety/efficacy. J Liposome Res.

[95] Watanabe A, Matsumoto K, Igari H, Uesato M, Yoshida S, Nakamura Y (2010). Comparison between concentrations of amphotericin B in infected lung lesion and in uninfected lung tissue in a patient treated with liposomal amphotericin B (AmBisome). Int J Infect Dis.

[96] Hoenigl M, Lewis R, van de Veerdonk FL, Verweij PE, Cornely OA. Liposomal amphotericin B—the future. J Antimicrob Chemother. 2022;77(Supplement_2):ii21–ii34. 10.1093/jac/dkac353 PMC9693803 36426674

[97] Bassetti M, Marchetti M, Chakrabarti A, Colizza S, Garnacho-Montero J, Kett DH (2013). A research agenda on the management of intra-abdominal candidiasis: results from a consensus of multinational experts. Intensive Care Med.

[98] Roy R, Tiwari M, Donelli G, Tiwari V (2018). Strategies for combating bacterial biofilms: a focus on anti-biofilm agents and their mechanisms of action. Virulence.

[99] Carrillo-Muñoz AJ, Finquelievich J, Tur-Tur C, Eraso E, Jauregizar N, Quindós G (2014). Combination antifungal therapy: a strategy for the management of invasive fungal infections. Rev Esp Quimioter.

[100] Meletiadis J, Andes DR, Lockhart SR, Ghannoum MA, Knapp CC, Ostrosky-Zeichner L (2022). Multicenter collaborative study of the interaction of antifungal combinations against *Candida* spp. by loewe additivity and bliss independence-based response surface analysis. J Fungi (Basel)..

[101] Khalifa HO, Majima H, Watanabe A, Kamei K (2021). In vitro characterization of twenty-one antifungal combinations against echinocandin-resistant and -susceptible *Candida glabrata*. J Fungi (Basel).

[102] Caballero U, Eraso E, Quindós G, Jauregizar N (2021). In vitro interaction and killing-kinetics of amphotericin B combined with anidulafungin or caspofungin against *Candida auris*. Pharmaceutics.

[103] Jaggavarapu S, Burd EM, Weiss DS (2020). Micafungin and amphotericin B synergy against *Candida auris*. Lancet Microbe.

[104] Fioriti S, Brescini L, Pallotta F, Canovari B, Morroni G, Barchiesi F (2022). Antifungal combinations against *Candida* species: from bench to bedside. J Fungi (Basel).

[105] Wong PN, Lo KY, Tong GMW, Chan SF, Lo MW, Mak SK (2008). Treatment of fungal peritonitis with a combination of intravenous amphotericin B and oral flucytosine, and delayed catheter replacement in continuous ambulatory peritoneal dialysis. Perit Dial Int.

[106] Wirth F, Ishida K (2020). Antifungal drugs: an updated review of central nervous system pharmacokinetics. Mycoses.

[107] Felton T, Troke PF, Hope WW (2014). Tissue penetration of antifungal agents. Clin Microbiol Rev.

[108] Pham CD, Iqbal N, Bolden CB, Kuykendall RJ, Harrison LH, Farley MM (2014). Role of FKS mutations in *Candida glabrata*: MIC values, echinocandin resistance, and multidrug resistance. Antimicrob Agents Chemother.

[109] Beyda ND, John J, Kilic A, Alam MJ, Lasco TM, Garey KW (2014). FKS mutant *Candida glabrata*: risk factors and outcomes in patients with candidemia. Clin Infect Dis.

[110] Roberts JA, Sime F, Lipman J, Hernández-Mitre MP, Baptista JP, Brüggemann RJ (2023). A protocol for an international, multicentre pharmacokinetic study for Screening Antifungal Exposure in Intensive Care Units: the SAFE-ICU study. Crit Care Resusc.

[111] Lin X, Hu X, Tang Z, Guo P, Liu X, Liang T (2023). Pharmacokinetics of voriconazole in peritoneal fluid of critically ill patients. Antimicrob Agents Chemother.

